# Association Between Midwall Late Gadolinium Enhancement and Sudden Cardiac Death in Patients With Dilated Cardiomyopathy and Mild and Moderate Left Ventricular Systolic Dysfunction

**DOI:** 10.1161/CIRCULATIONAHA.116.026910

**Published:** 2017-05-30

**Authors:** Brian P. Halliday, Ankur Gulati, Aamir Ali, Kaushik Guha, Simon Newsome, Monika Arzanauskaite, Vassilios S. Vassiliou, Amrit Lota, Cemil Izgi, Upasana Tayal, Zohya Khalique, Colin Stirrat, Dominique Auger, Nilesh Pareek, Tevfik F. Ismail, Stuart D. Rosen, Ali Vazir, Francisco Alpendurada, John Gregson, Michael P. Frenneaux, Martin R. Cowie, John G. F. Cleland, Stuart A. Cook, Dudley J. Pennell, Sanjay K. Prasad

**Affiliations:** From National Institute for Health Research Cardiovascular Biomedical Research Unit and Cardiovascular Magnetic Resonance Unit (B.P.H., A.G., A.A., M.A., V.S.V., A.L. C.I., U.T. Z.K., D.A., F.A., J.G.F.C., S.A.C., D.J.P., S.K.P.), Department of Cardiology (K.G., N.P., S.D.R., A.V., M.R.C.), Royal Brompton Hospital, London, United Kingdom; National Heart and Lung Institute, Imperial College, London, United Kingdom (B.P.H., A.A., K.G., V.S.V., A.L., U.T., S.D.R., A.V., F.A., M.R.C., J.G.F.C., S.A.C., D.J.P., S.K.P.); London School of Hygiene and Tropical Medicine, United Kingdom (S.N., J.G.); Norwich Medical School, University of East Anglia, United Kingdom (V.S.V., M.P.F); Centre for Cardiovascular Science, University of Edinburgh, United Kingdom (C.S.); King’s College London and Department of Cardiology, Guy’s and St Thomas’ Hospital, London, United Kingdom (T.F.I.); Department of Cardiology, Ealing Hospital, London, United Kingdom (S.D.R.); and National Heart Centre Singapore (S.A.C.).

**Keywords:** cardiovascular MRI, dilated cardiomyopathy, implantable cardioverter-defibrillator, late gadolinium enhancement, sudden cardiac death

## Abstract

**Background:**

Current guidelines only recommend the use of an implantable cardioverter defibrillator in patients with dilated cardiomyopathy for the primary prevention of sudden cardiac death (SCD) in those with a left ventricular ejection fraction (LVEF) <35%. However, registries of out-of-hospital cardiac arrests demonstrate that 70% to 80% of such patients have an LVEF >35%. Patients with an LVEF >35% also have low competing risks of death from nonsudden causes. Therefore, those at high risk of SCD may gain longevity from successful implantable cardioverter defibrillator therapy. We investigated whether late gadolinium enhancement (LGE) cardiovascular magnetic resonance identified patients with dilated cardiomyopathy without severe LV systolic dysfunction at high risk of SCD.

**Methods:**

We prospectively investigated the association between midwall LGE and the prespecified primary composite outcome of SCD or aborted SCD among consecutive referrals with dilated cardiomyopathy and an LVEF ≥40% to our center between January 2000 and December 2011 who did not have a preexisting indication for implantable cardioverter defibrillator implantation.

**Results:**

Of 399 patients (145 women, median age 50 years, median LVEF 50%, 25.3% with LGE) followed for a median of 4.6 years, 18 of 101 (17.8%) patients with LGE reached the prespecified end point, compared with 7 of 298 (2.3%) without (hazard ratio [HR], 9.2; 95% confidence interval [CI], 3.9–21.8; *P*<0.0001). Nine patients (8.9%) with LGE compared with 6 (2.0%) without (HR, 4.9; 95% CI, 1.8–13.5; *P*=0.002) died suddenly, whereas 10 patients (9.9%) with LGE compared with 1 patient (0.3%) without (HR, 34.8; 95% CI, 4.6–266.6; *P*<0.001) had aborted SCD. After adjustment, LGE predicted the composite end point (HR, 9.3; 95% CI, 3.9–22.3; *P*<0.0001), SCD (HR, 4.8; 95% CI, 1.7–13.8; *P*=0.003), and aborted SCD (HR, 35.9; 95% CI, 4.8–271.4; *P*<0.001). Estimated HRs for the primary end point for patients with an LGE extent of 0% to 2.5%, 2.5% to 5%, and >5% compared with those without LGE were 10.6 (95% CI, 3.9–29.4), 4.9 (95% CI, 1.3–18.9), and 11.8 (95% CI, 4.3–32.3), respectively.

**Conclusions:**

Midwall LGE identifies a group of patients with dilated cardiomyopathy and an LVEF ≥40% at increased risk of SCD and low risk of nonsudden death who may benefit from implantable cardioverter defibrillator implantation.

**Clinical Trial Registration:**

URL: http://clinicaltrials.gov. Unique identifier: NCT00930735.

## Abstract 2

Supplemental Digital Content is available in the text.

**Editorial, see p 2116**

Guidelines only recommend the use of implantable cardioverter defibrillators (ICDs) in patients with dilated cardiomyopathy (DCM) for the primary prevention of sudden cardiac death (SCD) in those with a left ventricular ejection fraction (LVEF) <35%.^1,2^ However, registries of out-of-hospital cardiac arrests demonstrate that 70% to 80% of such patients have a LVEF >35%, indicating that, in fact, the major burden of SCD occurs in patients with less severe degrees of LV impairment.^3,4^ The need to identify the subgroup of patients with mild and moderate reductions in LVEF at high risk of SCD has been highlighted by guidelines and statements from the American Heart Association, American College of Cardiology, European Society of Cardiology, and Heart Rhythm Societies.^2,5–7^ It is important to note that such patients are likely to have a lower risk of death from competing causes and fewer symptoms compared with patients with lower LVEF and may potentially have more to gain in terms of quality-adjusted life years from successful ICD therapy. This finding is particularly pertinent after the DANISH trial, which highlighted the importance of selecting patients with a low risk of death from other causes.^8^

Late gadolinium enhancement cardiovascular magnetic resonance (LGE-CMR) has shown that ≈30% of patients with DCM have midwall LGE, which represents replacement fibrosis, and that this provides incremental prognostic information to LVEF.^9–17^ Whether midwall LGE also identifies a high risk of SCD in patients with DCM and less severe reductions in LVEF, who might consequently benefit from an ICD, is unknown.^18^ Accordingly, we investigated whether midwall LGE is associated with SCD and aborted SCD in a large cohort of consecutive patients with DCM and LVEF ≥40%. A LVEF cutoff of ≥40% on CMR was chosen because this approximates to an LVEF of 35% on echocardiography, the current arbiter of primary prevention ICD implantation.^1,2,19–21^

## Methods

**Figure 1.**
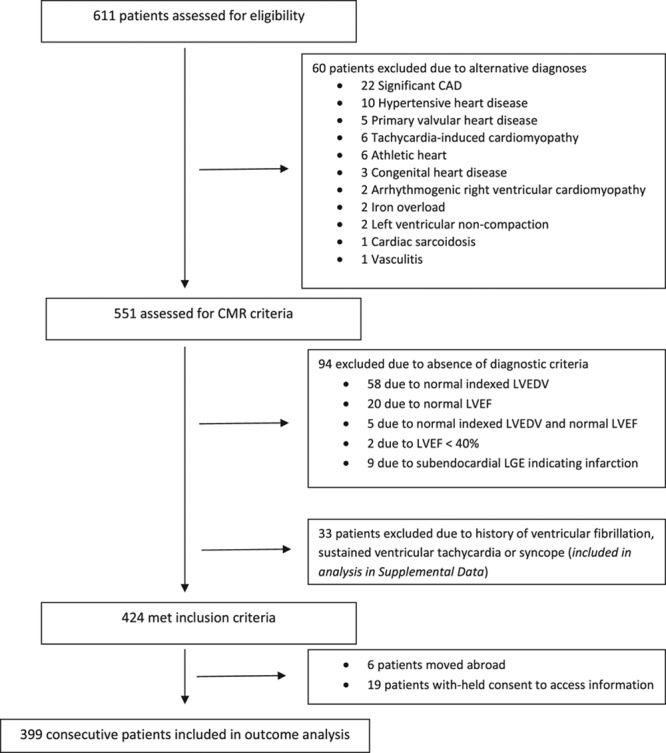
**Identification of the study population.** Flow chart detailing the identification, inclusion, and exclusion of patients. CAD indicates coronary artery disease; CMR, cardiovascular magnetic resonance; LGE, late gadolinium enhancement; LVEDV, left ventricular end-diastolic volume; and LVEF, left ventricular ejection fraction.

Patients seen in our cardiomyopathy service or referred for CMR assessment between November 2000 and December 2011 with DCM and an LVEF ≥40% were prospectively identified at the time of the scan and entered in a registry. Of 399 patients, 193 were included in a previous study of all-comers with DCM investigating LGE and all-cause mortality regardless of LVEF.^9^ These patients underwent extended follow-up for the current stand-alone, focused investigation in this select population. All participants provided informed consent, and the study was approved by the National Research Ethics Service. The inclusion criterion was a diagnosis of DCM confirmed using the World Health Organization/International Society and Federation of Cardiology criteria on the basis of an elevated left ventricular end-diastolic volume indexed to body surface area and reduced LVEF, compared with published age- and gender-specific reference values.^22^ Exclusion criteria are listed in Figure 1 and included the presence of significant coronary artery disease (CAD), defined as a stenosis of >50% in a major coronary artery, infiltrative disease, or valvular cardiomyopathy. To ensure that patients with ischemic aetiologies were not included, those individuals with infarct patterns of LGE were also excluded.^23^ Patients with a history of sustained ventricular tachycardia (VT), ventricular fibrillation, or syncope were excluded given a potential preexisting secondary prevention indication for ICD implantation. These patients have been included in an additional analysis in Figure I in the online-only Data Supplement). No patients had a preexisting indication for ICD implantation on the basis of primary prevention of SCD.

CMR was carried out on 1.5 Tesla scanners (Sonata/Avanto, Siemens) using a standardized protocol (online-only Data Supplement). The presence and location of midwall LGE were assessed by 2 independent Society of Cardiovascular Magnetic Resonance level 3-accredited operators blinded to clinical outcomes, with a third providing adjudication if necessary (MA, CI, FA). LGE was considered present if midmyocardial or subepicardial and visible in 2 phase-encoding directions and 2 orthogonal planes. The mass of LGE (grams) was quantified by a blinded operator using the full-width at half-maximum technique (CMR42, Circle Cardiovascular Imaging Inc) and indexed as a percentage of LV mass (MA, CI).

The prespecified primary end point was a composite of SCD or aborted SCD. SCD was defined as unexpected death ≤1 hour of the onset of cardiac symptoms in the absence of progressive cardiac deterioration, during sleep, or ≤24 hours of last being seen alive.^24^ Aborted SCD was defined as an appropriate ICD shock for ventricular arrhythmia, successful resuscitation after ventricular fibrillation, or sustained VT causing hemodynamic compromise and requiring cardioversion.^25^ The principal secondary end point was all-cause mortality. Additional secondary end points were (1) a composite of cardiovascular mortality (SCD, heart failure [HF], stroke, or thromboembolism), cardiovascular hospitalization, or cardiac transplantation; and (2) a HF composite of HF death, unplanned HF hospitalization, or cardiac transplantation. Death was attributed to HF if preceded by progressive deterioration in symptoms and signs. HF hospitalization was defined as an admission with new or worsening signs and symptoms of HF requiring intensification of HF-specific treatment.^24^

Patients were followed up throughout the study by postal questionnaire or telephone interview, through family physicians, clinics, and hospital notes. The duration of follow-up was calculated from the baseline scan until an end point occurred or last patient contact. Specifically, for the primary end point, any patients meeting the prespecified criteria for an event were censored from that date. A committee of cardiologists who were blinded to CMR data adjudicated outcomes (VV, AL, UT, ZK, DA, NP, AV). Deaths were also identified using the UK Health and Social Care Information Service to ensure none were missed. The adjudication committee established cause of death from death certification, postmortem results, and medical records using the American College of Cardiology/American Heart Association guidance.^24^ Aborted SCD was confirmed from records including ICD electrograms when necessary.

### Statistical Analysis

Baseline characteristics among those with and without LGE were compared using the Mann-Whitney U test for continuous data or Fisher exact test for categorical data. Kaplan-Meier survival curves were generated and compared using the log-rank test. Event times were measured from the baseline CMR date for ≤8 years. The associations between end points and the presence of LGE were analyzed using uni- and multivariable proportional hazard models. Results are presented as hazard ratios (HRs) with 95% confidence intervals (CIs). The multivariable model adjusted for these covariates: LVEF, New York Heart Assocation (NYHA) class, and age. As part of a sensitivity analysis, the univariable model was also adjusted using inverse-probability weighting by a propensity score, taking into account 13 baseline covariates, including the presence or absence of an ICD, allowing time-varying weights for this during follow-up. Details and full results of the propensity score analysis can be found in Tables I and II and Figure II in the online-only Data Supplement. To examine the dose-response relationship between LGE extent and the primary end point, estimated HRs were calculated for 4 groups depending on the extent of LGE: (1) no LGE, (2) 0% to 2.5%, (3) 2.5% to 5%, and (4) >5% of total LV mass using univariable proportional hazard models. We did not report estimates per 1% increase in LGE because of a clear nonlinear relationship between LGE extent and the primary end point. The percentage extent of LGE giving the largest c-statistic for the prediction of the primary end point was calculated from 1000 bootstrap samples. The C-statistic measured the degree to which a model can distinguish between cases and controls, taking values between 0.5 and 1.0, with larger values indicating better discrimination. To estimate the incremental predictive power of LGE above and beyond LVEF, a predicted 5-year risk of the primary end point was calculated from a Cox proportional model, which included LGE and categories of LVEF (40% to 43%, 44% to 47%, 48% to 51%, 52% to 55%, and 56% to 59%).

For comparison of participants with and without LGE, the sample size was estimated to provide >90% power to detect a significant difference in the primary end point if the true hazard ratio was ≥3. Statistical analyses were performed using Stata version 14 (StatCorp; SN and JG performed analyses). A *P*-value of <0.05 was taken as significant.

## Results

At baseline, 424 patients met the inclusion criteria, of which 25 either withheld consent for follow-up or had moved abroad (Figure 1). The report therefore focuses on 399 patients, of whom 145 were women, the median LVEF was 50% (interquartile range: 46% to 54%), and midwall LGE was present in 25.3%. Disagreement on the presence of LGE occurred in 8 cases, requiring adjudication by a third reviewer. Median follow-up until an event or last contact was 4.6 years (interquartile range: 3.5–7.0).

Baseline characteristics are presented in Table 1. Patients with midwall LGE were older (*P*=0.03) and more likely to be men (*P*<0.001), have diabetes mellitus (*P*=0.015) and receive loop diuretics (*P*=0.009). They also had lower heart rates (*P*=0.02) and diastolic blood pressure (*P*=0.02). The most common clinical presentation was with signs or symptoms of HF (n= 176; 44.1%). An additional 69 (17.2%) patients presented with symptoms of palpitation secondary to atrial arrhythmia or ventricular ectopy, 7 (1.8%) with symptoms of light-headedness or presyncope and 3 (0.8%) with first-degree atrioventricular block or a blunted chronotropic response. A further 39 (9.8%) patients were diagnosed after referral for family screening. Common indications classified as Other included diagnostic uncertainty or an abnormal ECG, such as the finding of left-bundle-branch block.

**Table 1.**
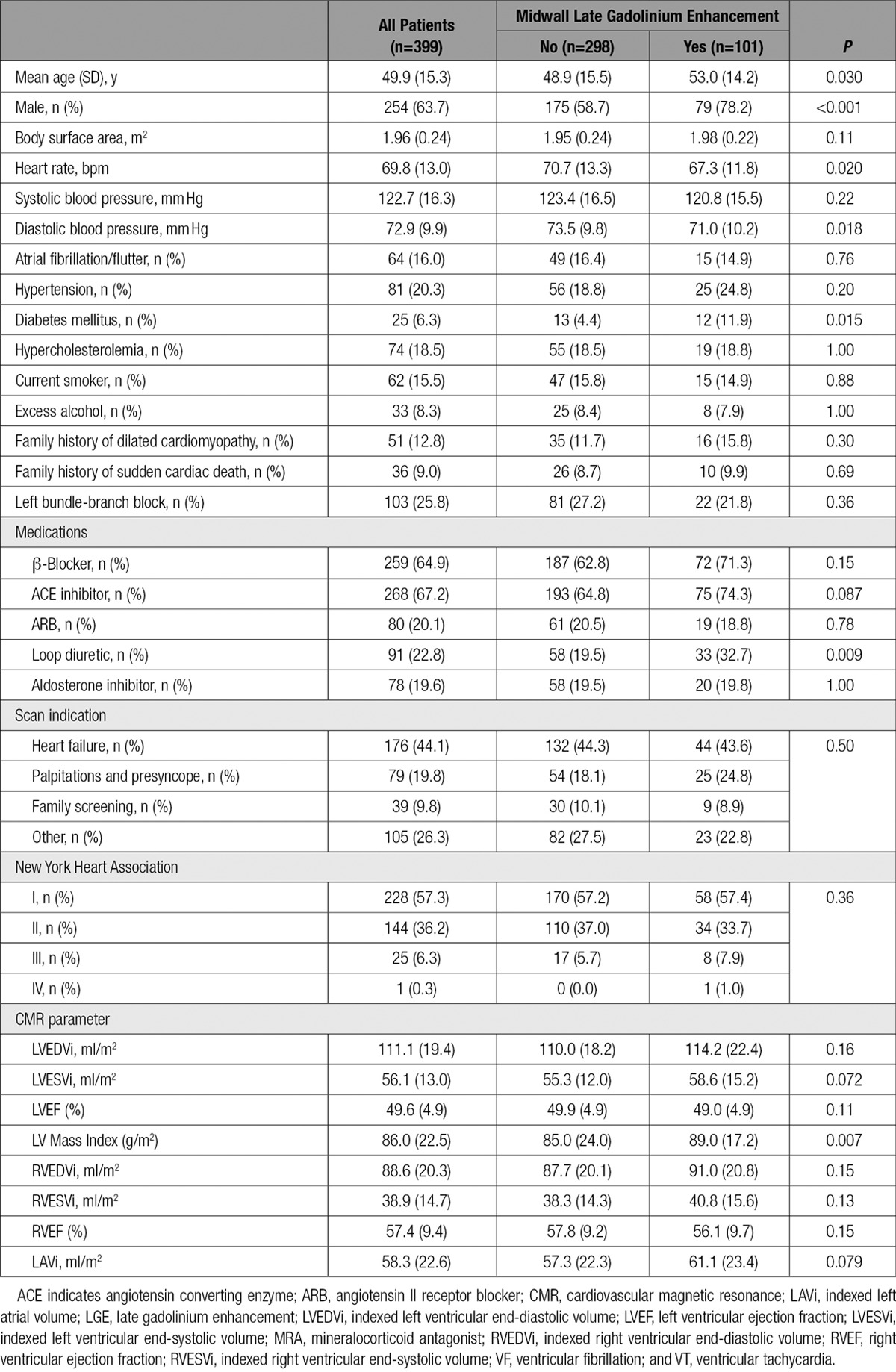
Baseline Demographics for Patients Based on the Presence or Absence of Midwall Late Gadolinium Enhancement

In line with guidelines, an ischemic etiology was considered in all patients and excluded as follows.^23^ All patients underwent LGE-CMR, and those with infarct patterns of enhancement were excluded.^23^ In addition, 268 (67.1%) patients underwent invasive or computed tomography coronary angiography, and a further 41 (10.3%) had perfusion imaging (nuclear or CMR) or stress echocardiography, with no provocation of ischemia. Of the remaining, 60 (15.0%) were ≤40 years of age without a history of angina or a family history of premature CAD, and further investigation was deemed unnecessary. All of the remaining 30 (7.5%) patients were free of angina and considered to have a low risk of CAD; in the absence of a class 1 indication, this was not performed.^23^ It is important to note that none of the patients underwent coronary revascularization or suffered an acute coronary syndrome during the follow-up period.

### Primary End Point: Sudden Cardiac Death and Aborted Sudden Cardiac Death

**Figure 2.**
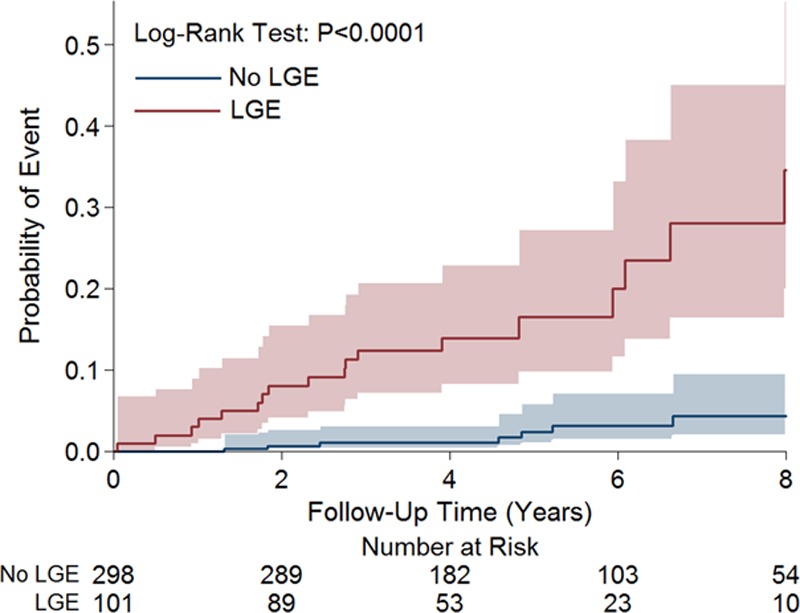
**Primary end point survival analysis.** Kaplan-Meier curve of the time to first event for the primary end point by presence (**red line**) or absence (**blue line**) of midwall late gadolinium enhancement (LGE).

During follow-up, 18 of 101 patients (17.8%) with LGE reached the primary end point compared with 7 of 299 patients (2.3%) without (HR, 9.2; 95% CI, 3.9–21.8; *P*<0.0001) (Figure 2). After adjusting for LVEF, NYHA class, and age, the presence of LGE predicted SCD and aborted SCD (HR, 9.3; 95% CI, 3.9–22.2; *P*<0.0001) (Table 2). The results were qualitatively the same after adjustment based on the propensity score (Table II in the online-only Data Supplement). There was little evidence of a dose-response relationship between LGE extent and the primary end point. Estimated HRs for patients with an LGE extent of 0% to 2.5%, 2.5% to 5%, and >5% were 10.6 (95% CI, 3.9–29.4), 4.9 (95% CI, 1.3–18.9), and 11.8 (95% CI, 4.3–32.3), respectively. In keeping with this relationship, the cutoff percentage extent of LGE that provided the largest c-statistic was >0% (95% CI, 0.0–8.5; c-statistic, 0.72).

**Table 2.**
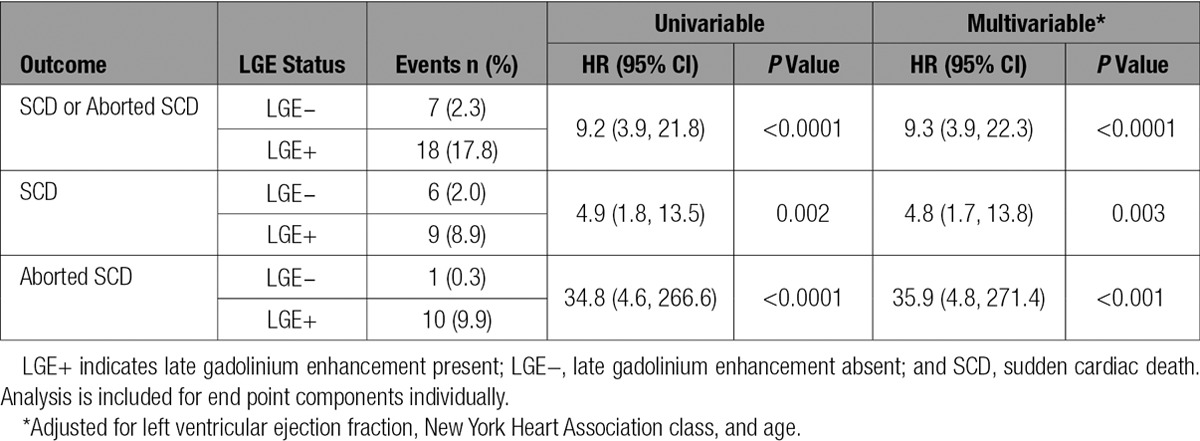
Univariable and Multivariable Analyses for the Primary End Point

Overall, 9 of 101 patients (8.9%) with LGE and 6 of 299 (2.0%) without died suddenly (HR, 4.9; 95% CI, 1.8–13.5; *P*=0.002). Correspondingly, 10 of 101 patients (9.9%) with LGE compared with 1 out of 299 patients (0.3%) without (HR, 34.8; 95% CI, 4.6–266.6; *P*<0.0001) suffered aborted SCD. After adjusting for LVEF, NYHA class, and age, the presence of LGE predicted SCD (HR, 4.8; 95% CI, 1.7–13.8; *P*=0.003) and aborted SCD (HR, 35.9; 95% CI, 4.8–271.4; *P*<0.001) when analyzed individually (Table 2). The results were qualitatively the same after adjustment based on the propensity score (Table II in the online-only Data Supplement).

**Figure 3.**
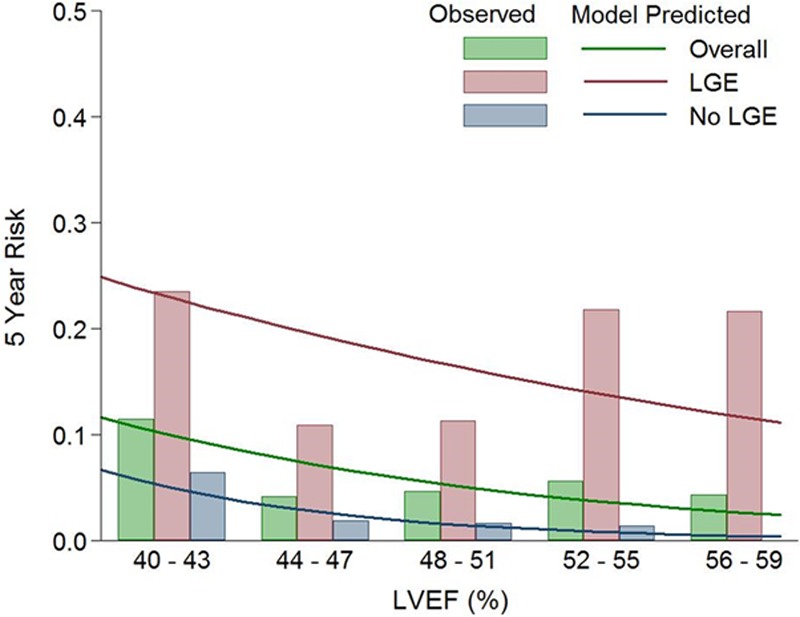
**Five-year risk estimates of the primary end point.** Five-year risk estimates for primary end point based on LVEF alone (**green line**) and midwall LGE status in addition to LVEF (**red line**, presence of LGE; **blue line**, absence of LGE). LGE indicates late gadolinium enhancement; and LVEF, left ventricular ejection fraction.

The predicted 5-year risk of aborted and actual SCD using a model including both LGE and LVEF was markedly different than a model using LVEF alone (Figure 3). For example, a patient with an LVEF of 45% had a 5-year predicted risk of 7.8% on the basis of LVEF alone, which fell to 3.2% in the absence of LGE but increased to 20.2% if LGE was present.

During follow-up, 32 patients (9.0%) had an ICD implanted before the occurrence of the primary end point, 17 of whom also received cardiac resynchronization therapy. Eighteen patients received ICDs in line with primary prevention guideline recommendations after deterioration in LVEF from baseline, 2 after new episodes of sustained VT without haemodynamic compromise, and 12 outside of conventional guideline recommendations after review at multidisciplinary meetings.^1,2^ Out of the latter 12 patients, 1 had a pathogenic lamin A/C mutation, 2 had a pacing indication with nonsustained VT, 3 had nonsustained VT and a family history of SCD, 4 had a history of nonsustained VT alone, and 2 presented with worsening HF and left bundle-branch block and had cardiac resynchronization therapy with a defibrillator. Of 32 patients who received an ICD system, 4 patients (23.5%) with and 0 patients (0.0%) without LGE had aborted sudden deaths. Of 367 patients without an ICD system, 9 patients (10.7%) with and 6 patients (2.1%) without LGE died suddenly.

### Secondary End Points

#### All-Cause Mortality

During follow-up, 32 deaths occurred, of which 19 were cardiovascular and 13 were not (cancer, end-stage lung disease, sepsis, and acute small bowel obstruction). The overall mortality rate was higher in patients with LGE (12.9% versus 6.4%; HR, 2.3; 95% CI, 1.1–4.6; *P*=0.02) (Figure III in the online-only Data Supplement). After adjustment for LVEF, NYHA class, and age, a trend toward higher mortality in those patients with LGE was noted; however, this did not reach statistical significance (HR, 2.0; 95% CI, 1.0–4.1; *P*=0.056).

#### Cardiovascular Death, Hospitalization, and Transplantation

There were 19 cardiovascular deaths (including 15 SCDs and 3 HF deaths) and 42 unplanned cardiovascular hospitalizations. Two patients underwent cardiac transplantation, 1 of whom had full histopathologic examination of the explanted heart. The gross and microscopic examinations correlated with LGE-CMR images (Figure IV in the online-only Data Supplement). Overall, this composite end point was more common in patients with LGE compared with those without (30.7% versus 10.7%; HR, 3.6; 95% CI, 2.2–5.8; *P*<0.0001) (Figure III in the online-only Data Supplement). After adjusting for LVEF, NYHA class, and age, the presence of LGE remained an independent predictor of the cardiovascular composite end point (HR, 3.2; 95% CI, 1.9–5.4; *P*<0.0001).

#### HF Death, HF Hospitalization, and Transplantation

There were 3 deaths secondary to HF and 18 unplanned HF admissions. The incidence of this composite end point was nominally more common in those with LGE compared with those without, although the difference was not statistically significant (7.9% versus 4.4%; HR, 1.9; 95% CI, 0.8–4.6; *P*=0.15) (Figure III in the online-only Data Supplement). This remained the case after adjustment for LVEF, NYHA class, and age (HR, 1.7; 95% CI, 0.7–4.2; *P*=0.27).

## Discussion

This large study in a population of well-treated and well-characterized DCM patients with mild or moderate LV impairment is the first investigation to demonstrate that midwall LGE on CMR is associated with a 9-fold increased risk of SCD and aborted SCD in this select subgroup. It is important to note that none of the patients within the cohort had a preexisting indication for ICD implantation at baseline, demonstrating the incremental value of LGE-CMR in risk stratification in this population. This focused investigation emphasizes the importance of extending risk stratification beyond LVEF assessment and extends earlier observations in HF populations, including both ischemic and nonischemic etiologies.^12,26^ Prediction of SCD and aborted SCD was independent of established prognostic variables, including LVEF, NYHA class, and age and qualitatively the same after adjustment for a large number of covariates based on a propensity score.

International guidelines and statements have highlighted the need to identify those patients with an LVEF >35% at highest risk of SCD because the major burden of SCD lies within this subgroup and is currently not accounted for by primary prevention ICD guidelines.^3–7^ Furthermore, as we move to an era of precision medicine, an expanding cohort of patients are being identified with milder reductions in LVEF in whom optimal therapy remains unclear.^27^ The DANISH trial has reemphasized the need to refine our current approaches to risk stratification.^8^ Although the trial demonstrated a reduction in SCD in patients with severely reduced LVEF randomized to ICD implantation, this finding was not associated with a significant reduction in all-cause mortality because of high rates of nonsudden cardiac death and noncardiac death.^8^ In other words, in this population of sick patients, ICD therapy simply changed the mode of death but not the overall mortality rate. This outcome illustrates the importance of selecting patients with a high risk of SCD and a low risk of nonsudden death who will be exposed to longer periods at risk of arrhythmias and may therefore have the most to gain from ICD therapy. Indeed in subgroup analysis of the DANISH trial, those patients most likely to benefit from ICD therapy were those at low risk of nonsudden death, specifically patients <59 years of age and those with an N-terminal pro-brain natriuretic peptide <1177pg/mL.^8^ Patients with mild or moderate reductions in LVEF have a low risk of nonsudden death and are also less likely to have limiting HF symptoms compared with those with more severe LV impairment and may therefore have the potential to gain a greater number of quality-adjusted life years after an aborted SCD. Our new data suggest a role for LGE-CMR in the identification of patients with less severe LV impairment who are at high risk of SCD and low risk of nonsudden death and who may therefore benefit from ICD implantation.

In patients with an LVEF ≥40%, over a median follow-up of 4.6 years, the risk of the primary end point in those with midwall LGE was 17.8%. In a similarly designed study with marginally longer follow-up (median 5.3 years), the risk of SCD and aborted SCD in all-comer DCM patients with an LVEF ≤35% was 17.9%, increasing to 27.9% in the subgroup with LGE, but dropping to only 11.1% in those without LGE.^9^ We have therefore observed an approximately equivalent rate of SCD events in patients with an LVEF ≥40% and LGE compared with all those with an LVEF ≤35%. This observation provides support for the CMR-Guide (NCT01918215) randomized trial, which aims to evaluate the benefit of ICD therapy in patients with LVEF 36% to 50% and LGE.

The greatest increment in SCD risk occurred between patients with no LGE and those with the smallest extent (0% to 2.5%). This finding was confirmed by analysis of Harrell’s c-statistic, which demonstrated an LGE extent cutoff of >0% as the best discriminator of event-free survival time. The lack of a linear dose-response relationship between the extent of LGE and the primary end point is novel and suggests that binary risk models based on the presence or absence of LGE are appropriate rather than models that examine risk based on the extent of LGE that assume linearity.^9,16^

Myocardial fibrosis is a widely accepted substrate for ventricular arrhythmia, supporting the biological plausibility of the findings. An electromapping study in patients with DCM demonstrated LGE in all patients with inducible VT or a history of sustained VT and mapped the arrhythmia to the corresponding location.^28^ In addition, areas of fibrosis interacting with channels of healthy myocardium in the peripheral heterogeneous zone of the scar have been associated with reentry wavefronts and targeting of these at catheter ablation reduces VT.^29–32^ It is therefore conceivable that the surface area of the gray zone between scar and healthy tissue determines the risk of VT, rather than the mass of the scar, explaining the lack of a dose-dependent association between LGE extent and SCD events in our study.^17,18^

### Limitations

This study was performed in a single, large-volume, experienced center. Although this enables the use of a standardized protocol and scan interpretation from the same independent operators, it introduces the possibility of referral bias. We do, however, report similar baseline characteristics to other registries.^13,33^ Moreover, the referral base is broad, from specialist and nonspecialist centers, and we report a range of common indications for the scan. Data from 193 of 399 patients were included in an earlier investigation on all-comers with DCM.^9^ These patients had extended follow-up in this study, which is unique in examining a focused clinical question in a targeted population using an alternative prespecified primary end point to address an unmet clinical need.

We also recognize the modest number of events in the study. We specified strict criteria for the primary end point, excluding appropriate anti-tachycardia pacing, to generate the most clinically meaningful data. Within this large study, we have identified a strong predictor of clinically important events responsible for a major burden of SCD in the DCM population. Based on the event rates in this study, a randomized trial of defibrillator therapy versus medical therapy in patients with an LVEF >40% and midwall LGE followed up for 5 years would require 971 patients to have 80% power to detect a difference in all-cause mortality at a significance level of 5%, assuming a 60% reduction in SCD with the intervention. This is comparable to the sample size of other large device trials.^8^

In this study, CAD was not excluded in all cases by coronary angiography. However, LGE-CMR has been shown to be as accurate in the diagnosis of the etiology of HF.^23^ In addition, the majority of patients who did not undergo coronary angiography were ≤40 years of age without a history of angina or a family history of premature CAD. Only 30 patients, all without a history of angina, were >40 years of age and had no additional investigations to exclude CAD. None of the patients suffered an acute coronary syndrome or had coronary revascularization during the study. Although we accept that CAD cannot be definitively excluded in this small group, significant CAD is nevertheless unlikely. The small size of this group means that this is unlikely to have biased the data to a significant extent.

ICD implantation was more frequent in patients with LGE; however, our results were consistent after adjusting for this as part of the propensity score analysis (Table II in the online-only Data Supplement). Although it is possible that the higher rate of ICD implantation reflects selection bias, the presence of LGE was not cited as an indication for implantation in any case. Among patients who had an ICD implanted, the rate of aborted SCD was higher in those with LGE compared with those without. Furthermore, despite the higher rate of ICD implantation in those with LGE, these patients had a higher rate of SCD. We acknowledge the limitations of aborted SCD as an end point and recognize that a proportion of arrhythmias resulting in appropriate shocks may have terminated spontaneously. However, our data on the association with SCD add robustness. We also recognize that a proportion of SCDs may relate to aneurysmal rupture and cerebral hemorrhage; however, in the absence of a biologically plausible link between LGE and these events, the effect would be to dilute the association between LGE and SCD rather than enhance it. ICD programming was at the discretion of the individual units. We did not routinely measure B-type natriuretic peptide, but we have included alternative variables that strongly predict prognosis in HF, such as indexed left atrial volume and NYHA class. Contemporary CMR techniques such as T1 mapping were not available at the outset, but we note a lack of consistency in the findings of other studies investigating its role in outcome prediction, with little evidence of incremental value in addition to LGE.^34,35^

## Conclusion

For the first time, we demonstrate that in patients with DCM and mild or moderate LV systolic impairment who do not meet conventional criteria for an ICD, the presence of midwall LGE identifies a subgroup at high risk of SCD. The risk of SCD in this subgroup was comparable to that seen in all-comer patients with an LVEF <35%; it is important to note that their risk of nonsudden cardiac death was low, suggesting that ICD therapy may have the potential to reduce all-cause mortality and extend quality life.

## Acknowledgments

The authors thank Dr Jan Lukas Robertus for providing images of histology.

## Sources of Funding

The work was supported by the National Institute for Health Research Cardiovascular Biomedical Research Unit at Royal Brompton and Harefield National Health Service Foundation Trust and Imperial College London. Dr Halliday is supported by a British Heart Foundation Clinical Research Training Fellowship (FS/15/29/31492). Dr Gulati received funding from the Coronary Artery Disease Research Association and Rosetrees Trust. Dr Prasad has received funding from the British Heart Foundation, the Medical Research Council, the Coronary Artery Disease Research Association, Rosetrees, and the Alexander Jansons Foundation.

## Disclosures

Dr Guha has received honoraria or travel assistance and speaker’s fees from Servier Laboratories, Pfizer, Boston Scientific, Medtronic, St Jude Medical, and Biotronik. Dr Frenneaux has received funding from Medtronic for investigator-initiated research. Dr Cowie has received research funding and speakers fees from Medtronic, Boston Scientific, and St Jude Medical. Dr Cleland sits on advisory boards for Medtronic and Sorin. Dr Cook has consulted for Illumina. Dr Pennell has received research support from Siemens and is a stockholder and director of Cardiovascular Imaging Solutions. Dr Prasad has received speaking fees from Bayer. The other authors report no conflicts of interest.

## Supplementary Material

## Clinical Perspective

### What Is New?

- This study demonstrates that midwall late gadolinium enhancement identifies patients with dilated cardiomyopathy and mild and moderate reductions in left ventricular ejection fraction at high risk of sudden cardiac death.

### What Are the Clinical Implications?

- Patients with dilated cardiomyopathy, midwall late gadolinium enhancement, and mild or moderate reductions in left ventricular ejection fraction should be recognized as having a high risk of sudden cardiac death.
- This finding is important because these patients are not currently offered implantable cardioverter defibrillators for the primary prevention of sudden cardiac death on the basis of guideline recommendations.
- Because of low competing risks of death from nonsudden causes, it is possible that these patients will benefit from implantable cardioverter defibrillator implantation.
- Randomized trials investigating the benefit of pharmacological therapies and implantable cardioverter defibrillator implantation in patients with late gadolinium enhancement and less severe reduction in left ventricular ejection fraction are now required.

## References

[1] Russo AM, Stainback RF, Bailey SR, Epstein AE, Heidenreich PA, Jessup M, Kapa S, Kremers MS, Lindsay BD, Stevenson LW (2013). ACCF/HRS/AHA/ASE/HFSA/SCAI/SCCT/SCMR 2013 appropriate use criteria for implantable cardioverter-defibrillators and cardiac resynchronization therapy.. J Am Coll Cardiol.

[2] Priori SG, Blomstrom-Lundqvist C, Mazzanti A, Blom N, Borggrefe M, Camm J, Elliott PM, Fitzsimons D, Hatala R, Hindricks G, Kirchhof P, Kjeldsen K, Kuck KH, Hernandez-Madrid A, Nikolaou N, Norekval TM, Spaulding C, Van Veldhuisen DJ (2015). 2015 ESC guidelines for the management of patients with ventricular arrhythmias and the prevention of sudden cardiac death.. Eur Heart J.

[3] Gorgels AP, Gijsbers C, de Vreede-Swagemakers J, Lousberg A, Wellens HJ (2003). Out-of-hospital cardiac arrest–the relevance of heart failure: the Maastricht Circulatory Arrest Registry.. Eur Heart J.

[4] Stecker EC, Vickers C, Waltz J, Socoteanu C, John BT, Mariani R, McAnulty JH, Gunson K, Jui J, Chugh SS (2006). Population-based analysis of sudden cardiac death with and without left ventricular systolic dysfunction: two-year findings from the Oregon Sudden Unexpected Death Study.. J Am Coll Cardiol.

[5] Goldberger JJ, Cain ME, Hohnloser SH, Kadish AH, Knight BP, Lauer MS, Maron BJ, Page RL, Passman RS, Siscovick D, Stevenson WG, Zipes DP (2008). American Heart Association/American College of Cardiology Foundation/Heart Rhythm Society scientific statement on noninvasive risk stratification techniques for identifying patients at risk for sudden cardiac death.. Heart Rhythm.

[6] Fishman GI, Chugh SS, Dimarco JP, Albert CM, Anderson ME, Bonow RO, Buxton AE, Chen PS, Estes M, Jouven X, Kwong R, Lathrop DA, Mascette AM, Nerbonne JM, O’Rourke B, Page RL, Roden DM, Rosenbaum DS, Sotoodehnia N, Trayanova NA, Zheng ZJ (2010). Sudden cardiac death prediction and prevention: report from a National Heart, Lung, and Blood Institute and Heart Rhythm Society Workshop.. Circulation.

[7] Zipes DP, Camm AJ, Borggrefe M, Buxton AE, Chaitman B, Fromer M, Gregoratos G, Klein G, Moss AJ, Myerburg RJ, Priori SG, Quinones MA, Roden DM, Silka MJ, Tracy C, Smith SC, Jacobs AK, Adams CD, Antman EM, Anderson JL, Hunt SA, Halperin JL, Nishimura R, Ornato JP, Page RL, Riegel B, Blanc JJ, Budaj A, Dean V, Deckers JW, Despres C, Dickstein K, Lekakis J, McGregor K, Metra M, Morais J, Osterspey A, Tamargo JL, Zamorano JL (2006). ACC/AHA/ESC 2006 guidelines for management of patients with ventricular arrhythmias and the prevention of sudden cardiac death.. Circulation.

[8] Køber L, Thune JJ, Nielsen JC, Haarbo J, Videbæk L, Korup E, Jensen G, Hildebrandt P, Steffensen FH, Bruun NE, Eiskjær H, Brandes A, Thøgersen AM, Gustafsson F, Egstrup K, Videbæk R, Hassager C, Svendsen JH, Høfsten DE, Torp-Pedersen C, Pehrson S, DANISH Investigators (2016). Defibrillator implantation in patients with nonischemic systolic heart failure.. N Engl J Med.

[9] Gulati A, Jabbour A, Ismail TF, Guha K, Khwaja J, Raza S, Morarji K, Brown TD, Ismail NA, Dweck MR, Di Pietro E, Roughton M, Wage R, Daryani Y, O’Hanlon R, Sheppard MN, Alpendurada F, Lyon AR, Cook SA, Cowie MR, Assomull RG, Pennell DJ, Prasad SK (2013). Association of fibrosis with mortality and sudden cardiac death in patients with nonischemic dilated cardiomyopathy.. JAMA.

[10] Assomull RG, Prasad SK, Lyne J, Smith G, Burman ED, Khan M, Sheppard MN, Poole-Wilson PA, Pennell DJ (2006). Cardiovascular magnetic resonance, fibrosis, and prognosis in dilated cardiomyopathy.. J Am Coll Cardiol.

[11] Gao P, Yee R, Gula L, Krahn AD, Skanes A, Leong-Sit P, Klein GJ, Stirrat J, Fine N, Pallaveshi L, Wisenberg G, Thompson TR, Prato F, Drangova M, White JA (2012). Prediction of arrhythmic events in ischemic and dilated cardiomyopathy patients referred for implantable cardiac defibrillator: evaluation of multiple scar quantification measures for late gadolinium enhancement magnetic resonance imaging.. Circ Cardiovasc Imaging.

[12] Klem I, Weinsaft JW, Bahnson TD, Hegland D, Kim HW, Hayes B, Parker MA, Judd RM, Kim RJ (2012). Assessment of myocardial scarring improves risk stratification in patients evaluated for cardiac defibrillator implantation.. J Am Coll Cardiol.

[13] Kuruvilla S, Adenaw N, Katwal AB, Lipinski MJ, Kramer CM, Salerno M (2014). Late gadolinium enhancement on cardiac magnetic resonance predicts adverse cardiovascular outcomes in nonischemic cardiomyopathy: a systematic review and meta-analysis.. Circ Cardiovasc Imaging.

[14] Lehrke S, Lossnitzer D, Schöb M, Steen H, Merten C, Kemmling H, Pribe R, Ehlermann P, Zugck C, Korosoglou G, Giannitsis E, Katus HA (2011). Use of cardiovascular magnetic resonance for risk stratification in chronic heart failure: prognostic value of late gadolinium enhancement in patients with non-ischaemic dilated cardiomyopathy.. Heart.

[15] Müller KA, Müller I, Kramer U, Kandolf R, Gawaz M, Bauer A, Zuern CS (2013). Prognostic value of contrast-enhanced cardiac magnetic resonance imaging in patients with newly diagnosed non-ischemic cardiomyopathy: cohort study.. PLoS One.

[16] Neilan TG, Coelho-Filho OR, Danik SB, Shah RV, Dodson JA, Verdini DJ, Tokuda M, Daly CA, Tedrow UB, Stevenson WG, Jerosch-Herold M, Ghoshhajra BB, Kwong RY (2013). CMR quantification of myocardial scar provides additive prognostic information in nonischemic cardiomyopathy.. J Am Coll Cardiol. Cardiovasc Imaging.

[17] Disertori M, Rigoni M, Pace N, Casolo G, Masè M, Gonzini L, Lucci D, Nollo G, Ravelli F (2016). Myocardial fibrosis assessment by LGE is a powerful predictor of ventricular tachyarrhythmias in ischemic and nonischemic LV dysfunction: a meta- analysis.. J Am Coll Cardiol. Cardiovasc Imaging.

[18] Bilchick KC (2016). The fault is in our scars: LGE and ventricular arrhythmia risk in LV dysfunction.. J Am Coll Cardiol. Cardiovasc Imaging.

[19] Hoffmann R, von Bardeleben S, ten Cate F, Borges AC, Kasprzak J, Firschke C, Lafitte S, Al-Saadi N, Kuntz-Hehner S, Engelhardt M, Becher H, Vanoverschelde JL (2005). Assessment of systolic left ventricular function: a multi-centre comparison of cineventriculography, cardiac magnetic resonance imaging, unenhanced and contrast-enhanced echocardiography.. Eur Heart J.

[20] Malm S, Frigstad S, Sagberg E, Larsson H, Skjaerpe T (2004). Accurate and reproducible measurement of left ventricular volume and ejection fraction by contrast echocardiography: a comparison with magnetic resonance imaging.. J Am Coll Cardiol.

[21] Ponikowski P, Voors AA, Anker SD, Bueno H, Cleland JG, Coats AJ, Falk V, Gonzalez-Juanatey JR, Harjola VP, Jankowska EA, Jessup M, Linde C, Nihoyannopoulos P, Parissis JT, Pieske B, Riley JP, Rosano GM, Ruilope LM, Ruschitzka F, Rutten FH, van der Meer P (2016). 2016 ESC guidelines for the diagnosis and treatment of acute and chronic heart failure.. Eur J Heart Fail.

[22] Maceira AM, Prasad SK, Khan M, Pennell DJ (2006). Normalized left ventricular systolic and diastolic function by steady state free precession cardiovascular magnetic resonance.. J Cardiovasc Magn Reson.

[23] Assomull RG, Shakespeare C, Kalra PR, Lloyd G, Gulati A, Strange J, Bradlow WM, Lyne J, Keegan J, Poole-Wilson P, Cowie MR, Pennell DJ, Prasad SK (2011). Role of cardiovascular magnetic resonance as a gatekeeper to invasive coronary angiography in patients presenting with heart failure of unknown etiology.. Circulation.

[24] Hicks KA, Tcheng JE, Bozkurt B, Chaitman BR, Cutlip DE, Farb A, Fonarow GC, Jacobs JP, Jaff MR, Lichtman JH, Limacher MC, Mahaffey KW, Mehran R, Nissen SE, Smith EE, Targum SL (2015). 2014 ACC/AHA key data elements and definitions for cardiovascular endpoint events in clinical trials.. Circulation.

[25] Buxton AE, Calkins H, Callans DJ, DiMarco JP, Fisher JD, Greene HL, Haines DE, Hayes DL, Heidenreich PA, Miller JM, Poppas A, Prystowsky EN, Schoenfeld MH, Zimetbaum PJ, Goff DC, Grover FL, Malenka DJ, Peterson ED, Radford MJ, Redberg RF (2006). ACC/AHA/HRS 2006 key data elements and definitions for electrophysiological studies and procedures.. J Am Coll Cardiol.

[26] Cheong BY, Muthupillai R, Wilson JM, Sung A, Huber S, Amin S, Elayda MA, Lee VV, Flamm SD (2009). Prognostic significance of delayed-enhancement magnetic resonance imaging: survival of 857 patients with and without left ventricular dysfunction.. Circulation.

[27] Pinto YM, Elliott PM, Arbustini E, Adler Y, Anastasakis A, Böhm M, Duboc D, Gimeno J, de Groote P, Imazio M, Heymans S, Klingel K, Komajda M, Limongelli G, Linhart A, Mogensen J, Moon J, Pieper PG, Seferovic PM, Schueler S, Zamorano JL, Caforio AL, Charron P (2016). Proposal for a revised definition of dilated cardiomyopathy, hypokinetic non-dilated cardiomyopathy, and its implications for clinical practice: a position statement of the ESC working group on myocardial and pericardial diseases.. Eur Heart J.

[28] Bogun FM, Desjardins B, Good E, Gupta S, Crawford T, Oral H, Ebinger M, Pelosi F, Chugh A, Jongnarangsin K, Morady F (2009). Delayed-enhanced magnetic resonance imaging in nonischemic cardiomyopathy: utility for identifying the ventricular arrhythmia substrate.. J Am Coll Cardiol.

[29] de Bakker JM, Coronel R, Tasseron S, Wilde AA, Opthof T, Janse MJ, van Capelle FJ, Becker AE, Jambroes G (1990). Ventricular tachycardia in the infarcted, Langendorff-perfused human heart: role of the arrangement of surviving cardiac fibers.. J Am Coll Cardiol.

[30] Hsia HH, Marchlinski FE (2002). Electrophysiology studies in patients with dilated cardiomyopathies.. Card Electrophysiol Rev.

[31] Estner HL, Zviman MM, Herzka D, Miller F, Castro V, Nazarian S, Ashikaga H, Dori Y, Berger RD, Calkins H, Lardo AC, Halperin HR (2011). The critical isthmus sites of ischemic ventricular tachycardia are in zones of tissue heterogeneity, visualized by magnetic resonance imaging.. Heart Rhythm.

[32] Perez-David E, Arenal A, Rubio-Guivernau JL, del Castillo R, Atea L, Arbelo E, Caballero E, Celorrio V, Datino T, Gonzalez-Torrecilla E, Atienza F, Ledesma-Carbayo MJ, Bermejo J, Medina A, Fernández-Avilés F (2011). Noninvasive identification of ventricular tachycardia-related conducting channels using contrast-enhanced magnetic resonance imaging in patients with chronic myocardial infarction: comparison of signal intensity scar mapping and endocardial voltage mapping.. J Am Coll Cardiol.

[33] Merlo M, Stolfo D, Anzini M, Negri F, Pinamonti B, Barbati G, Ramani F, Lenarda AD, Sinagra G (2015). Persistent recovery of normal left ventricular function and dimension in idiopathic dilated cardiomyopathy during long-term follow-up: does real healing exist?. J Am Heart Assoc.

[34] Chen Z, Sohal M, Voigt T, Sammut E, Tobon-Gomez C, Child N, Jackson T, Shetty A, Bostock J, Cooklin M, O’Neill M, Wright M, Murgatroyd F, Gill J, Carr-White G, Chiribiri A, Schaeffter T, Razavi R, Rinaldi CA (2015). Myocardial tissue characterization by cardiac magnetic resonance imaging using T1 mapping predicts ventricular arrhythmia in ischemic and non-ischemic cardiomyopathy patients with implantable cardioverter-defibrillators.. Heart Rhythm.

[35] Puntmann VO, Carr-White G, Jabbour A, Yu CY, Gebker R, Kelle S, Hinojar R, Doltra A, Varma N, Child N, Rogers T, Suna G, Arroyo Ucar E, Goodman B, Khan S, Dabir D, Herrmann E, Zeiher AM, Nagel E, International T1 Multicentre CMR Outcome Study (2016). T1- mapping and outcome in nonischemic cardiomyopathy: all- cause mortality and heart failure.. JACC Cardiovasc Imaging.

