# Supplementary material for: Association Between Midwall Late Gadolinium Enhancement and Sudden Cardiac Death in Patients With Dilated Cardiomyopathy and Mild and Moderate Left Ventricular Systolic Dysfunction
Source: Circulation. 2017 May 30;135(22):2106–15. doi: 10.1161/CIRCULATIONAHA.116.026910 (PMC5444425; doi:10.1161/CIRCULATIONAHA.116.026910)
Supplement: Supplementary file 1 [file cir-135-2106-s001.pdf]

SUPPLEMENTAL MATERIAL

Supplemental Methods

CMR protocol

Steady-state free-precession sequences were used to acquire cine images in standard long axis planes and in contiguous short axis slices from the atrioventricular ring to the apex. Intravenous gadopentetate dimeglumine or gadobutrol (Schering, Berlin, Germany) were used at a dose of 0.1mmol/kg. An inversion recovery gradient echo sequence was used to obtain LGE images, ten minutes after gadolinium administration, in identical planes to the cine images, in two phase-encoding directions. Inversion times were optimized to null the myocardium. Ventricular volumes and mass were calculated using dedicated software (CMRtools, Cardiovascular Imaging Solutions, London, UK). Left atrial volumes indexed to body surface area (LAVi) were measured using the biplane area-length method[1].

Supplemental Primary End-point Analysis

We report the primary end-point analyses for those patients meeting the inclusion criteria set out in the main manuscript and in addition, those patients with a prior history of ventricular fibrillation, ventricular tachycardia and syncope, excluded from the analysis in the main manuscript. Overall, 432 pateints were followed-up for a median of 4.5 (IQR: 3.4 – 6.6) years, of whom 159 were women, the median LVEF was 50% (IQR:46-54%) and mid-wall LGE was present in 25.7%.

During follow-up, 21 of 111 patients (18.9%) with LGE reached the primary end-point compared to 11 of 321 patients (3.4%) without (HR 6.5; 95% CI 3.2-13.5; P<0.0001) (*Figure A)*. After adjusting for LVEF, NYHA class, age and gender the presence of LGE predicted SCD and aborted SCD (HR 7.6; 95%CI 3.3-17.4; p<0.0001).

Overall, 9 of 111 patients (8.1%) with and 7 of 321 patients (2.2%) without fibrosis died suddenly (HR 4.1; 95% CI 1.6-10.9; p=0.004). Correspondingly, 13 of 111 patients (11.7%) with fibrosis compared to 4 out of 321 patients (1.2%) without (HR 10.7; 95% CI 3.5-32.9; p<0.0001) suffered aborted SCD. Following adjustment, the presence of fibrosis predicted SCD (HR 3.5; 95% CI 1.1-10.8; p=0.03) and aborted SCD (HR 14.6; 95% CI 4.7-46.2; p<0.001) when analyzed individually.

Supplemental Tables

Supplemental Table 1. Propensity score model

	OR (95% CI)	p
LVEF (per 10)	0.94 (0.54, 1.62)	0.82
Age (per 10)	1.14 (0.94, 1.37)	0.18
Male	2.46 (1.34, 4.49)	0.003
LAVi (per 10)	1.01 (0.89, 1.15)	0.83
NYHA II	0.97 (0.54, 1.73)	0.55
NYHA III / IV	1.74 (0.61, 4.97)	
LVEDVi (per 10)	1.06 (0.90, 1.24)	0.50
RVEF (per 10)	0.94 (0.66, 1.33)	0.72
ACE Inhibitor	1.30 (0.74, 2.30)	0.36
Beta Blocker	1.34 (0.75, 2.37)	0.32
Diabetes	2.65 (1.06, 6.62)	0.037
HR (per 10)	0.89 (0.72, 1.09)	0.26
Scan Indication		
Heart Failure	1.00	0.24
Palpitation / Presyncope	1.29 (0.68, 2.45)	
Family Screening	1.50 (0.61, 3.68)	
Other	0.68 (0.35, 1.32)	
ICD Implant	3.31 (1.67, 6.58)	<0.001

Baseline covariates used to construct the propensity score model were as follows: LVEF, NYHA class, age, gender, LAVi, LVEDVi, RVEF, ACE inhibitor and beta-blocker prescription, heart rate, scan indication and history of diabetes mellitus. ICD implantation was also included, allowing time-varying weights during follow-up.

(ACE – angiotensin converting enzyme, ARB – angiotensin II receptor blocker, HR – heart rate, LAVi – indexed left atrial volume, LVEDVi – indexed left ventricular end-diastolic volume, LVEF – left ventricular ejection fraction, RVEF –right ventricular ejection fraction, VT – ventricular tachycardia, VF - ventricular fibrillation)

Supplemental Table 2. Results of the Propensity score analysis

Outcome	LGE Status	Events n (%)	IPW Estimate	
			HR (95% CI)	P Value
**SCD or Aborted SCD**	LGE -	7 (2.3)	8.0 (3.3, 19.5)	<0.0001
	LGE +	18 (17.8)		
**SCD**	LGE-	6 (2.0)	4.6 (1.6, 13.1)	0.005
	LGE+	9 (8.9)		
**Aborted SCD**	LGE-	1 (0.3)	32.9 (4.3, 249.9)	<0.001
	LGE+	10 (9.9)		
**All-Cause Mortality**	LGE-	19 (6.4)	2.0 (0.9, 4.2)	0.086
	LGE+	13 (12.9)		
**HF Death, Hospitalisation or Transplant**	LGE-	13 (4.4)	1.6 (0.6, 4.4)	0.32
	LGE+	8 (7.9)		
**CV Death, Hospitalisation or Transplant**	LGE-	32 (10.7)	3.1 (1.8, 5.4)	<0.0001
	LGE+	31 (30.7)		

Inverse probability weighting analyses for the primary and secondary end-points. (weights based on left and right ventricular ejection fraction, indexed left ventricular end-diastolic volume, New York Heart Association Class, age, gender, indexed left atrial volume, ACE inhibitor and beta-blocker prescription, heart rate, scan indication, history of diabetes mellitus and the presence or absence of an ICD allowing time carrying weights for the latter; CI – confidence intervals, CV – cardiovascular, HF – heart failure, IPW: inverse probability weighting, LGE+ – late gadolinium enhancement present, LGE- - late gadolinium enhancement absent; OR – odds ratio; SCD – sudden cardiac death)

Supplemental Figures & Figure Legends

Supplemental Figure 1. Supplemental primary end-point analysis

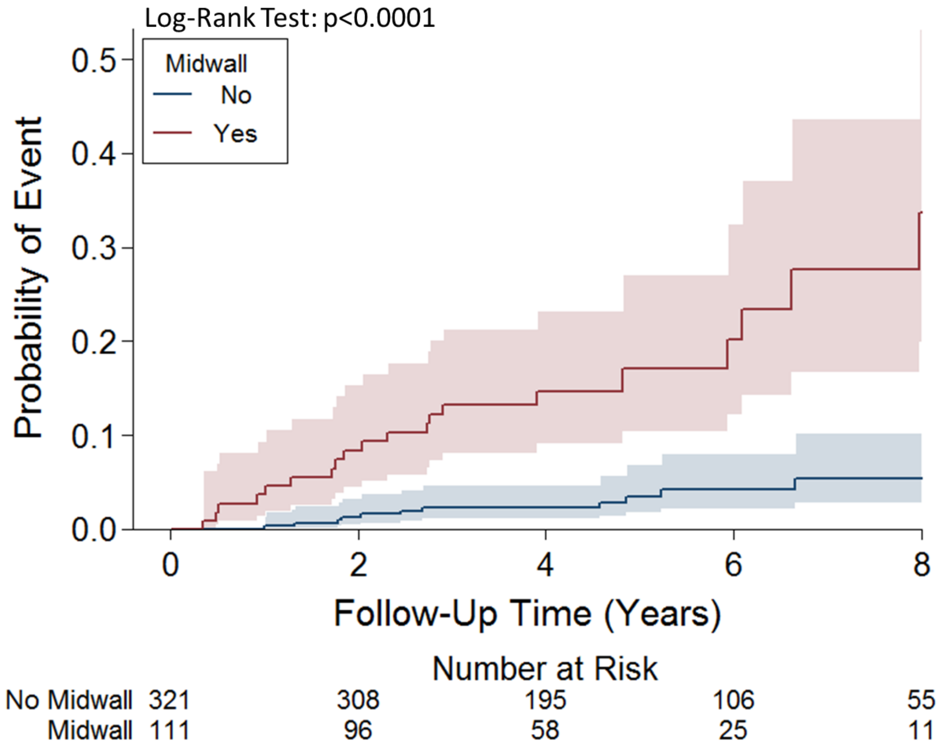

Kaplan-Meier curves of the time to first event for the primary end-points by presence (red-line) or absence (blue line) of mid-wall LGE, including patients with a prior history of sustained ventricular tachycardia, ventricular fibrillation or syncope.

Supplemental Figure 2. Histogram of the propensity score distribution in the groups with and without LGE

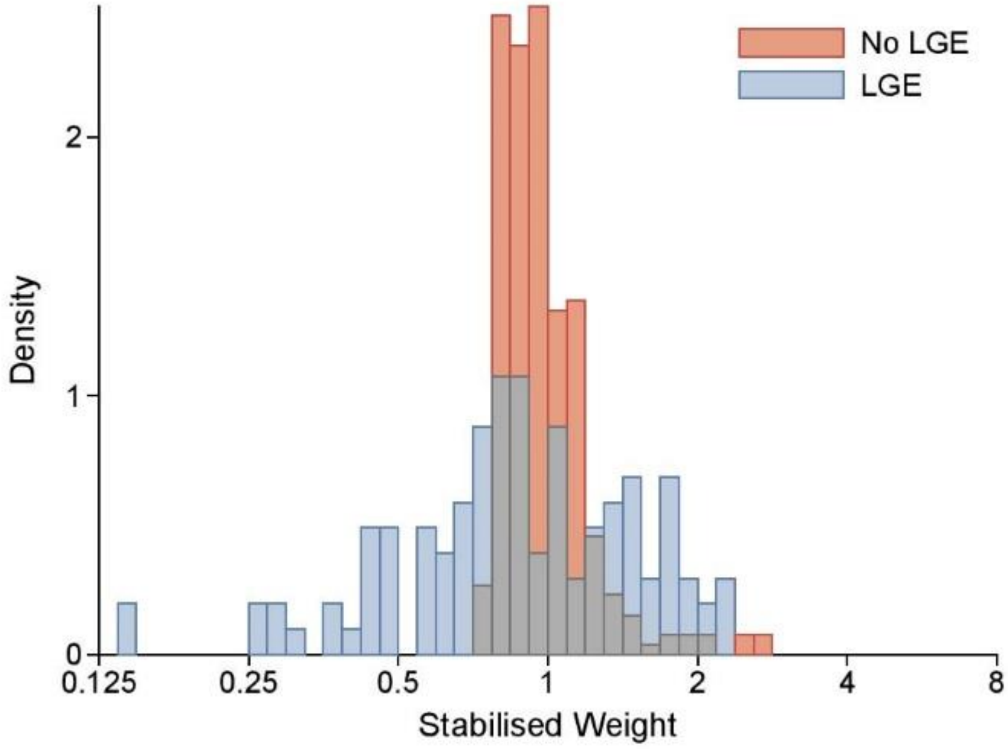

(LGE – late gadolinium enhancement)

Supplemental Figure 3. Secondary end-points

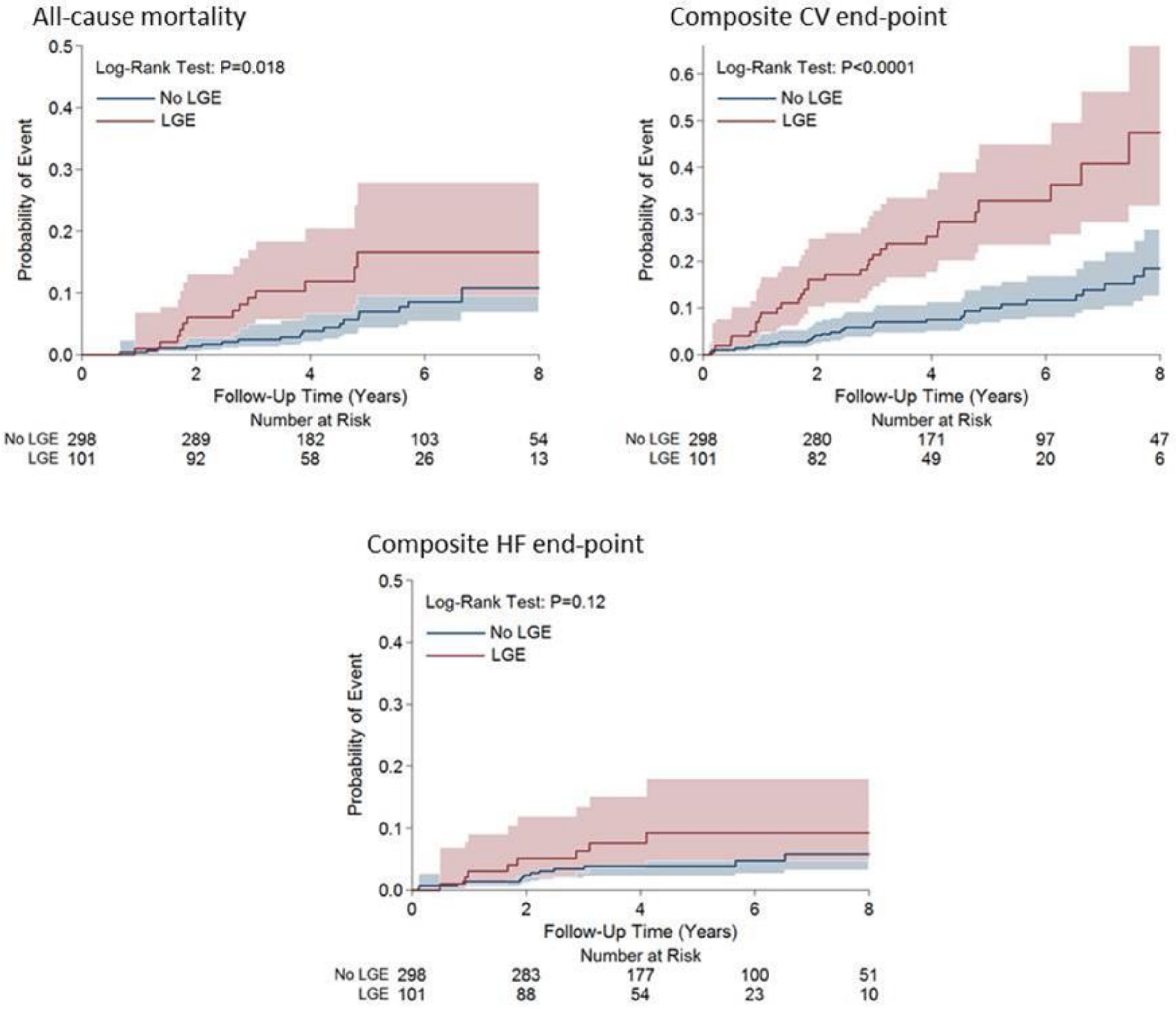

Kaplan-Meier curves of the time to first event for the secondary end-points by presence (red-line) or absence (blue line) of mid-wall LGE.

Supplemental Figure 4. Histological correlation

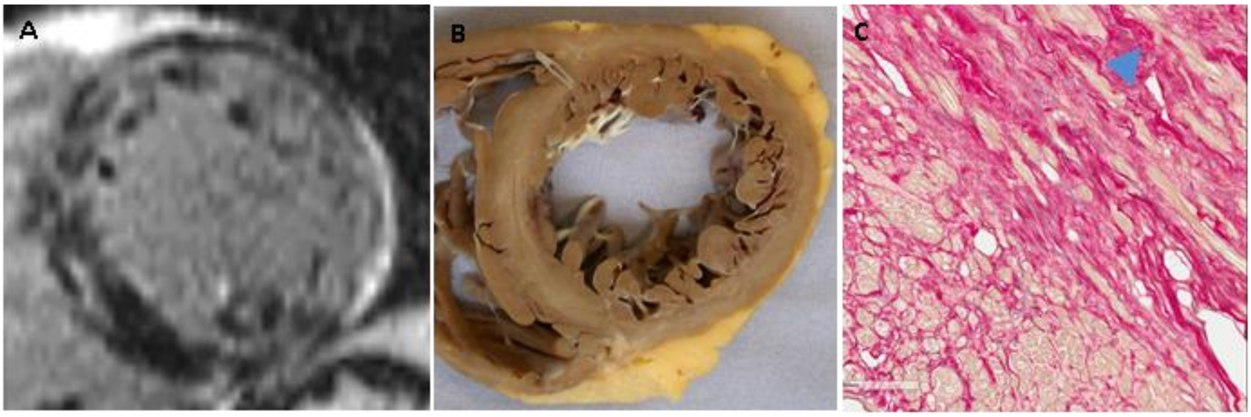

A: Pre-transplant late gadolinium enhancement (LGE) cardiovascular magnetic resonance demonstrating extensive mid-wall and sub-epicardial LGE, including the septum at mid-ventricular level. *B:* Post-transplant gross examination of a short-axis slice at mid-ventricular level confirming extensive mid-wall replacement fibrosis. *C:* Post-transplant micrscopic examination of a specimen from the septum of the explanted left ventricle, at x300 magnification, confirming replacement (arrow) and pericellular fibrosis.

References

1. Gulati A, Ismail TF, Jabbour A, Ismail NA, Morarji K, Ali A, Raza S, Khwaja J, Brown TD, Liodakis E, Baksi AL, Shakur R, Guha K, Roughton M, Wage R, Cook SA, Alpendurada F, Assomull RG, Mohiaddin RH, Cowie MR, Pennell DJ, Prasad SK. Clinical utility and prognostic value of left atrial volume assessment by cardiovascular magnetic resonance in non-ischaemic dilated cardiomyopathy. EurJ Heart Fail. 2013;15:660-670.
